# Genetic Analysis of Teosinte Alleles for Kernel Composition Traits in Maize

**DOI:** 10.1534/g3.117.039529

**Published:** 2017-02-10

**Authors:** Avinash Karn, Jason D. Gillman, Sherry A. Flint-Garcia

**Affiliations:** * Division of Plant Sciences, University of Missouri, Columbia, Missouri 65211; † United States Department of Agriculture-Agricultural Research Service, Columbia, Missouri 65211

**Keywords:** maize, teosinte, introgression population, kernel composition, quantitative trait loci (QTL), multi-parent populations, multiparental populations, MPP, Multiparent Advanced Generation Inter-Cross (MAGIC)

## Abstract

Teosinte (*Zea mays* ssp. *parviglumis*) is the wild ancestor of modern maize (*Zea mays* ssp. *mays*). Teosinte contains greater genetic diversity compared with maize inbreds and landraces, but its use is limited by insufficient genetic resources to evaluate its value. A population of teosinte near isogenic lines (NILs) was previously developed to broaden the resources for genetic diversity of maize, and to discover novel alleles for agronomic and domestication traits. The 961 teosinte NILs were developed by backcrossing 10 geographically diverse *parviglumis* accessions into the B73 (reference genome inbred) background. The NILs were grown in two replications in 2009 and 2010 in Columbia, MO and Aurora, NY, respectively, and near infrared reflectance spectroscopy and nuclear magnetic resonance calibrations were developed and used to rapidly predict total kernel starch, protein, and oil content on a dry matter basis in bulk whole grains of teosinte NILs. Our joint-linkage quantitative trait locus (QTL) mapping analysis identified two starch, three protein, and six oil QTL, which collectively explained 18, 23, and 45% of the total variation, respectively. A range of strong additive allelic effects for kernel starch, protein, and oil content were identified relative to the B73 allele. Our results support our hypothesis that teosinte harbors stronger alleles for kernel composition traits than maize, and that teosinte can be exploited for the improvement of kernel composition traits in modern maize germplasm.

Maize (*Zea mays* ssp. *mays*) is one of the most economically valuable grain crops in the world (Awika 2011). It is a significant resource for food, feed, and biofuel, and provides raw materials for various industrial applications. Maize was domesticated from teosinte (*Zea mays* ssp. *parviglumis*) in southern Mexico ∼7500–9000 years ago (Matsuoka *et al.* 2002; Piperno *et al.* 2009; Hufford *et al.* 2012) but bears striking morphological differences in terms of plant, inflorescence, and seed architecture (Doebley *et al.* 1995). Today, maize breeders and geneticists are well aware of the reduction in genetic diversity during crop domestication, especially in genes underlying traits that were targeted by the selection process (Flint-Garcia 2013), which resulted in lower or no variation in traits and limited the discovery of novel alleles that have potential to improve a crop’s germplasm (Flint-Garcia *et al.* 2009).

Teosinte has minute kernels compared with maize, enclosed within a hard, stony fruitcase, a trait not present in maize inbreds and landraces (Dorweiler *et al.* 1993). Similarly, kernel composition differs between teosinte and modern maize; on a dry matter basis (DMB), inbred maize kernels are ∼71.7% starch, ∼9.5% protein, and ∼4.3% oil (Watson *et al.* 2003). In contrast, teosinte kernels have ∼52.92% starch, ∼28.71% protein, and ∼5.61% oil, strongly suggesting that the increase in kernel size, fruitcase-less kernels, and increase in kernel starch were the targets of artificial selection during maize domestication (Flint-Garcia *et al.* 2009).

Recent sequencing efforts suggest that 2–4% of the maize genome was impacted due to the artificial selection process. There is a significant reduction in the genetic variation of genes underlying selected traits, whereas, the 96–98% of the neutral genes remain to retain high levels of genetic diversity (Wright *et al.* 2005; Hufford *et al.* 2012). One long-term goal of maize breeding is to transfer novel genetic variation from teosinte for the improvement of modern maize germplasm (Flint-Garcia *et al.* 2009).

A teosinte near isogenic population [hereafter referred to as teosinte near isogenic lines (NILs)] was developed to provide new genetic resources for complex trait dissection in maize, and identify and introduce novel genetic diversity from teosinte (Liu *et al.* 2016). NILs have a strong potential to identify and fine-map quantitative trait loci (QTL), and have been widely applied in several crop species, including maize (Graham *et al.* 1997; Szalma *et al.* 2007), soybean (Muehlbauer *et al.* 1991; Jiang *et al.* 2009), and tomato (Eshed and Zamir 1995; Brouwer and St. Clair 2004). Another advantage of NILs is the reduction of confounding “noise” from genetic background and epistatic interactions between QTL. These characteristics of NIL populations make them suitable genetic resources to fine-map and identify novel alleles for complex agronomic traits. Statistically, NILs are more accurate in estimating QTL effects because the phenotypic differences are caused only by allelic differences at the introgression sites (Kaeppler 1997).

In this study, we aimed to simultaneously discover and evaluate the potential of novel alleles from teosinte for improving the nutritional and kernel quality of modern maize germplasm.

## Materials and Methods

### Maize teosinte near isogenic libraries

The development and genotyping of the 10 teosinte NIL families (58–185 lines per family) was described previously (Liu *et al.* 2016). Briefly, the NILs were developed by backcrossing 10 accessions of geographically diverse *Z. mays* ssp. *parviglumis* into the inbred B73 for four generations prior to inbreeding, creating a total of 961 NILs. These NILs were genotyped via a GoldenGate assay (Illumina, San Diego, CA), and a subset of 728 out of the 1106 nested association mapping (NAM) markers were selected based on polymorphism between B73 and the 10 teosinte parents (McMullen *et al.* 2009; Liu *et al.* 2016). Genotypic data for the teosinte NILs can be accessed from the supplemental data in Liu *et al.* (2016). Genotypic ratios revealed by examining marker data shows that the BC_4_S_2_ teosinte NIL population averaged ∼95.9% homozygous B73, ∼2.6% heterozygous B73/teosinte, and ∼1.5% homozygous teosinte. An individual teosinte NIL had an average of 2.4 chromosomal segments from teosinte which, when combined, encompass ∼4% of the teosinte genome introgressed into a B73 background (Liu *et al.* 2016).

### Near infrared reflectance and nuclear magnetic resonance calibration for estimating kernel composition traits

Previously, kernel starch, protein, and oil content was estimated for 26,305 seed samples from seven grow-outs of the NAM population using a Perten Diode Array 7200 (DA7200) instrument (Perten Instruments, Stockholm, Sweden) and a proprietary (Syngenta Seeds, Inc.) near infrared reflectance (NIR) calibration (Cook *et al.* 2012). In order to calibrate our own local machines, we selected two sets of 210 and 45 seed samples from among these 26,305 samples, in order to span the wide range of values for starch protein and oil based on these Syngenta estimates. The original composition values based on the Syngenta calibration were used solely to choose samples with extreme values for calibration and are not used anywhere in the current study.

The 255 calibration samples were sent to the University of Missouri Experiment Station Chemical Laboratories for proximate analysis, following the official methods of AOAC International (2006). These reference values for starch, protein, and oil were then adjusted to a DMB and used in the calibration of our own machines. The reference samples had the following ranges: 55.3–82.3% for starch, 6.8–21.4% for protein, and 1.7–6.3% for oil (Table 1).

**Table 1:** Descriptive statistics of reference composition values on a DMB in the NIR and NMR calibration sets comprised of NAM samples

Trait	Instrument	*n*	Mean, %	Median, %	SD	Variance	Range, %
Starch	NIR	209	68.65	68.60	5.40	29.2	55.34–82.33
Protein	NIR	210	12.91	12.90	3.08	9.51	6.76–21.40
Oil	NMR	45	3.94	4.02	1.42	2.03	1.66–6.34

In the NIR calibration, intact kernels were scanned on a FOSS 6500 NIR instrument (FOSS North America, Eden Prairie, MN). Reflectance spectra (*R*) from bulk whole grains of at least 50 kernels from each sample were collected at 10-nm intervals in the NIR region from 400 to 2500 nm. Each sample was scanned five times and averaged. Absorbance values were calculated as log(1/*R*) using ISIscan and exported via WinISI IV software for regression analysis (Supplemental Material, Table S1). The collected NIR spectra of the samples were preprocessed using Savitzky–Golay first derivative as described by Spielbauer *et al.* (2009), and multiplicative scatter correction as described by Geladi *et al.* (1985). Spectral preprocessing and partial least squares (PLS) regression analysis were carried out using the UnScrambler version 6.11 (CAMO ASA, Trondheim, Norway).

A PLS1 regression method was used to derive calibration models for protein and starch, as well as oil (Baye *et al.* 2006). In the PLS1 regression analysis, preprocessed spectral data were used as descriptor data (*X* variable) and analytical data as response data set (*Y* variable). Initially, of the 210 samples with reference data, 190 samples were randomly chosen for NIR calibration and 20 samples for external validation (Table S2). The performance of the various regression models was evaluated based on the coefficient of correlation (*r*) between the reference and NIR-predicted values and SE of calibration (SEC) in the validation set of 20 samples (Table S2). Once a satisfactory calibration model was developed for each trait, the 20 samples from the validation set were added back to the calibration set in order to develop a final calibration model (NIR calibration equations for kernel protein and starch are provided in Table S3). The NIR oil calibration was poor (*r* = 0.63; SEC = 0.78) (Table S2), and was not used for the remainder of the study.

Similarly, a bench-top MQC analyzer (Oxford Instruments) nuclear magnetic resonance (NMR) instrument was calibrated with samples from 45 NAM recombinant inbred lines (RILs) with wide range of known analytical values for oil content (Cook *et al.* 2012) using the in-built calibration software. The NMR resonance values from each sample were collected in triplicate at the operating frequency 5 MHz from ∼10 g of intact maize kernels, which was regressed against the reference values to develop a model (Table S4). The performance of the NMR model to measure oil content was determined by the coefficient of correlation (*r*) and SE between the reference and NMR-predicted values.

### Phenotypic data collection and analysis in teosinte NILs

A total of 961 teosinte NIL entries were grown as a random complete block design and self-pollinated in two locations with two replications each: Columbia, MO and Aurora, NY in the year 2009 and 2010, respectively, with B73 as an experimental control. Kernel composition data (starch, protein, and oil) were obtained from bulk intact kernels from each plot using the NIR and NMR calibrations developed above.

Least square means across environments (Table S5) were calculated using PROC MIXED for individual kernel composition traits, and broad sense heritability (*H*^2^) was calculated by the method described in Holland *et al.* (2003) in SAS software version 9.2 (SAS Institute Inc., Cary, NC).

### Joint-linkage QTL analysis

A genetic map based on the NAM population was used for the joint-linkage QTL analysis following the protocol of Liu *et al.* (2016). Briefly, appropriate *P*-value thresholds (starch = 1.31 × 10^−06^, protein = 6.06 × 10^−07^, and oil = 1.12 × 10^−06^) for the joint-linkage mapping were determined by 1000 permutations in SAS. Joint step regression was conducted using PROC GLM SELECT, where the model contained a family main effect and marker effects nested within families (Cook *et al.* 2012). We used PROC GLM for the final model and to estimate additive effects of the teosinte alleles. The presence of significant additive effects of the teosinte alleles were determined by a *t*-test comparison of the parental means *vs.* the control B73 allele. QTL support intervals were calculated as a 1-LOD drop from the peak of the QTL.

### Data availability

The authors state that all data necessary for confirming the conclusions presented in the article are represented fully within the article.

### Results

The NIR models were successfully able to predict starch (*r* = 0.82, SEC = 2.7) and protein (*r* = 0.97, SEC = 0.72) (Table 2), while the oil model was unable to accurately predict oil (*r* = 0.63, SEC = 0.78) (Table S2). Instead, we developed an NMR model to predict oil content (*r* = 0.98, error = 0.09) (Table 3).

**Table 2:** Final NIR calibration statistics for starch and protein content on a DMB in intact maize kernels

Trait	Instrument	Spectral Range, nm	Spectra Treatment	*n*	*r*	SEC
Starch	FOSS 6500 NIR	410–2500	MSC; 1st Deri	210	0.82	2.70
Protein	FOSS 6500 NIR	900–2500	MSC	210	0.97	0.72

MSC, multiplicative scatter correction; 1st Deri, Savitzky–Golay first derivative.

**Table 3:** NMR calibration statistics for oil content on DMB in intact maize kernels

Trait	Instrument	Operating Frequency, MHz	*n*	Weight, g	*r*	SD	SE
Oil	Oxford Instruments NMR	5	45	∼10	0.98	0.30	0.09

**Figure 1:**
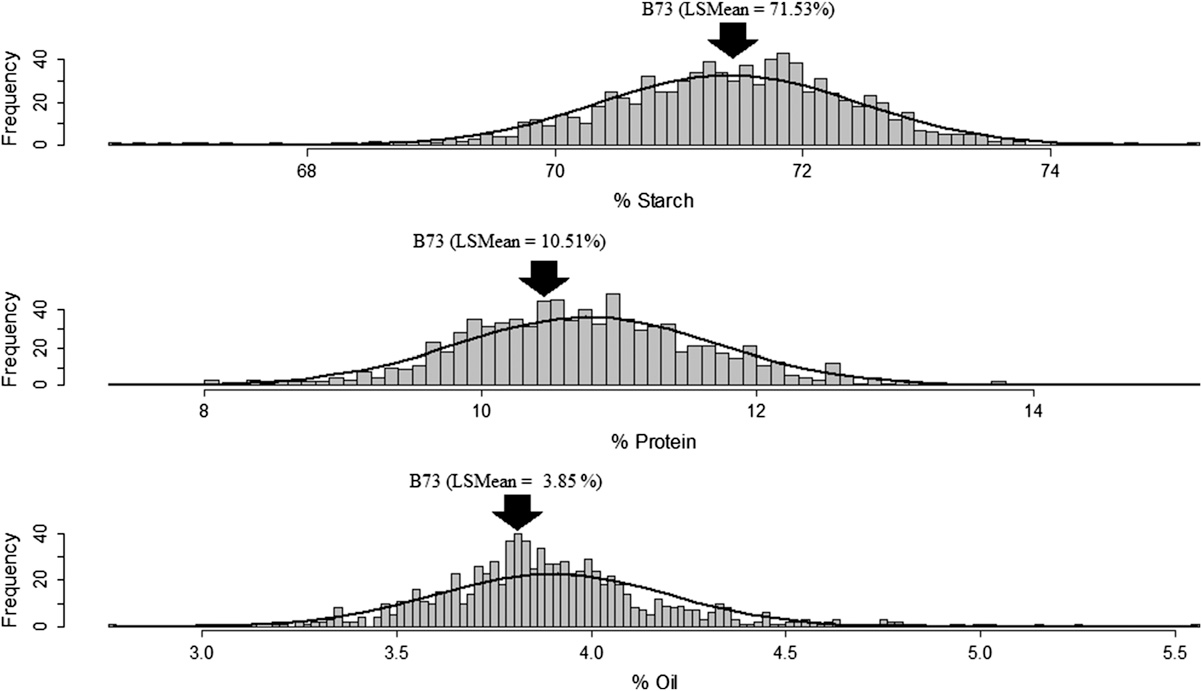
Distribution of kernel starch, protein, and oil content in the teosinte NILs. The least squares mean (LSMean) for B73 is indicated by a black arrow.

The NIR calibrations were used to predict starch and protein content and the NMR calibration was used to predict oil content in the teosinte NIL trial. Due to few or no kernels in some NIL samples, kernel composition data were obtained from 858 out of 961 teosinte NILs, and ranged from 66.4 to 75.1% for starch, 7.32 to 15.2% for protein, and 2.7 to 5.5% for oil (Figure 1 and Table 4). The distribution of the teosinte NILs was skewed in the direction of the expected teosinte allelic effect as predicted by teosinte composition phenotypes relative to maize: a longer tail for lower starch, and a longer tail in the direction of higher protein and oil.

**Table 4:** Descriptive statistics of predicted starch, protein, and oil content in teosinte NILs, and results for the joint-linkage QTL analysis for each trait

Trait	*n*	Mean, %	Range, %	Difference, %	*H*^2^	QTL	Marker (Chromosome)	*R*^2^, %
Starch	857	71.41	66.42–75.17	8.85	0.70	2	t251; PZA01962.12 (3)	18.0
							t643; PZA03057.3 (9)	
Protein	857	10.77	7.32–15.20	7.87	0.76	3	t50; PZA02070.1 (1)	23.1
							t254; PHM1675.29 (3)	
							t437; PZA03172.3 (5)	
Oil	858	3.89	2.77–5.55	2.78	0.94	6	t53; PZA02135.2 (1)	45.0
							t149; PZA01993.7 (2)	
							t254; PHM1675.29 (3)	
							t408; PZA01779.1 (5)	
							t476; PZA03461.1 (6)	
							t604; PZA00951.1 (8)	

Significant negative phenotypic correlations were detected between starch and protein (*r* = −0.823, *P* < 0.0001) and between starch and oil (*r* = −0.083, *P* < 0.01). A significant positive phenotypic correlation was detected between protein and oil (*r* = 0.11, *P* < 0.001). These correlations are in line with those previously observed in diverse maize germplasm (Cook *et al.* 2012), as well as QTL studies involving high-oil parents (Zhang *et al.* 2008). Broad-sense heritability for starch, protein and oil content in teosinte NILs were 70, 74, and 94%, respectively (Table 4).

**Figure 2:**
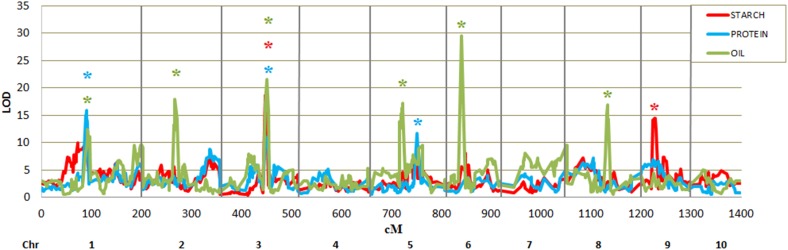
Joint-linkage QTL analysis for kernel starch, protein, and oil content in teosinte NILs. Horizontal units, cM; vertical units, log of odds (LOD). Asterisks indicate the presence of significant QTL for starch (red), protein (blue), or oil (green).

Joint stepwise regression identified a total of eight QTL across the three traits: two starch QTL that explained 18% of the variation, three protein QTL that explained 23% of the variation, and six oil QTL that explained 45% of variation (Figure 2, Table 4, and Table S6). The chromosome 1 QTL was significant for both protein and oil, and the chromosome 3 QTL was significant for all three traits.

**Figure 3:**
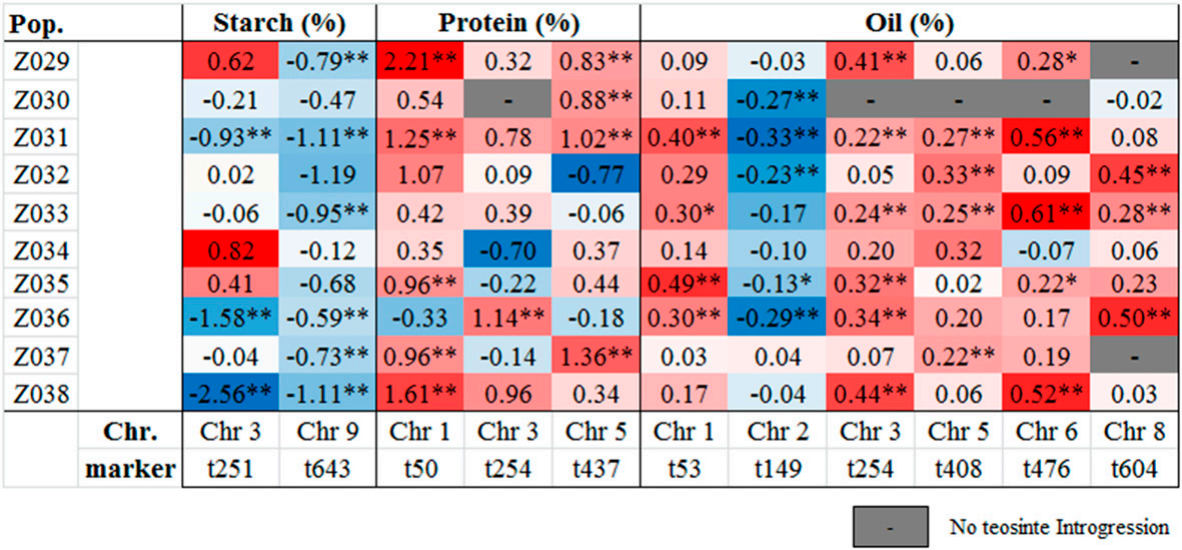
Heat map displaying additive effects of teosinte alleles across 10 populations for starch, protein, and oil content QTL relative to B73. The NIL population is indicated on the vertical axis and marker genotype associated with the QTL is indicated on the horizontal axis. Color and intensity reflect the direction and strength of the allelic effect: red represents teosinte alleles that increase the trait value and blue represents teosinte alleles that decrease the trait value. *, significant at *P* = 0.05; **, significant at *P* = 0.01; –, no teosinte introgression available for *t*-test.

As the 10 teosinte accessions were crossed to a common reference line (B73), it was possible to accurately estimate additive effects of the teosinte alleles relative to B73 and to each other. Each of the 10 teosinte NIL donors was allowed to have an independent allele by fitting a population-by-marker term in the stepwise regression and final models, as described by Buckler *et al.* (2009) and Cook *et al.* (2012). We identified a total of nine starch, 12 protein, and 25 oil teosinte alleles that were significant (Table 5 and Table S7) (*P* < 0.05). The direction of the allelic effects corresponded well with the skew of the phenotypes (Figure 1). Because teosinte has lower starch content and higher protein and oil than maize (Flint-Garcia *et al.* 2009), we anticipated that most of the teosinte alleles would decrease starch and increase protein and oil. In fact, all of the significant alleles were in the anticipated direction, with the exception of the oil QTL on chromosome 2, where all five of the significant alleles decreased oil. All the QTL had a range of strong additive allelic effects, with the largest allelic effects for starch, protein, and oil QTL being −2.56, 2.21, and 0.61% dry matter, respectively, and displayed both positive and negative additive allelic effects depending upon the trait (Figure 3).

**Table 5:** Comparing number of QTL and additive allelic effects of maize (NAM) and teosinte alleles for kernel composition traits

Trait	NAM Population			Teosinte NILs		
	QTL	Allelic Effects			Allelic Effects	
		Minimum, %	Maximum, %	QTL	Minimum, %	Maximum, %
Starch	21	−0.62	0.65	2	−2.56	0.82
Protein	26	−0.38	0.34	3	−0.77	2.21
Oil	22	−0.12	0.21	6	−0.33	0.61

## Discussion

The endosperm is the largest structure (80–85% of the kernel by weight) in maize kernels (fao 1992), and starch (∼71% by weight) and protein (∼11% by weight) are the major chemical components. In contrast, oil is only a minor constituent of the total kernel (∼4% of the kernel weight) but is major chemical component of the embryo/germ (10–12% of the kernel by weight) (fao 1992; Flint-Garcia *et al.* 2009). Kernel composition in maize is influenced by various environmental and genetic factors (Wilson *et al.* 2004), and has been the target of domestication and more recent breeding. Therefore, it is critical to understand what genes control these important traits, and to determine the levels of genetic diversity for these genes in order to continue the improvement of maize grain for food, feed, and fuel.

One aim in this study was to develop rapid, nondestructive phenotyping methods for kernel starch, protein, and oil in intact kernels of maize. We accomplished this by developing nondestructive, robust and high-throughput methods using NIR and NMR instrumentation. The calibration and validation results and PLS models revealed that NIR is capable of predicting kernel protein and starch content (Table 2), but unable to reliably predict oil (Table S2).

NIR can efficiently predict a higher number of kernel composition traits in ground samples than in intact kernels of maize. In ground samples, the kernel chemical components are evenly distributed throughout the sample. However, in intact seed, oil is nonuniformly distributed throughout the kernel. Because the oil is concentrated in the embryo, reflectance methods are highly sensitive to the directionality of the kernels in the sample (more embryos facing toward or away from the instrument). Because our goal was to nondestructively phenotype composition traits, we decided to explore an NMR-base method to characterize oil.

In previous studies, NMR has been used to predict oil content in both 25 g and single-kernel intact maize kernels with high accuracy (*r* > 0.99, error = 0.05) (Alexander *et al.* 1967). Our NMR model can predict oil content with less than half the amount of material (∼10 g) of intact maize kernels very accurately (*r* > 0.98, error = 0.09) in <15 sec. These parameters are important both for efficiency and to avoid inadvertent selection bias, as some of the lines in teosinte NILs produced <50 kernels.

Broad-sense heritability estimates for kernel starch and protein were moderate (70 and 76%, respectively), but extremely high for kernel oil (94%), which indicates that kernel oil content is more stable over environments than either kernel starch and protein. When compared with the NAM population, heritability for kernel protein and starch was lower in our teosinte NILs but higher for kernel oil content. Heritability in NIL populations is generally lower than RIL populations, likely because of lower genotypic variance in the near isogenic background than among RILs due to the uniformity in the lines (Eichten *et al.* 2011).

**Figure 4:**
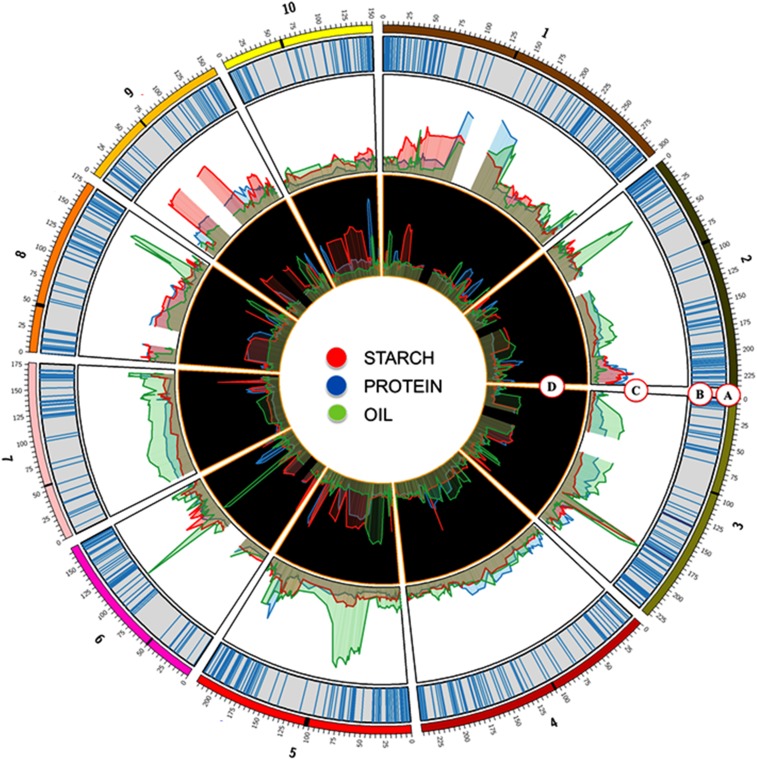
Circos plot displaying: (A) the 10 chromosomes of maize, (B) physical coordinates of the SNP markers, (C) joint-linkage QTL peaks in the teosinte NIL analysis, and (D) joint-linkage QTL peaks in the NAM analysis (Cook *et al.* 2012).

Cook *et al.* (2012) evaluated the maize NAM population for kernel starch, protein, and oil content. The NAM population was developed by crossing 25 diverse founder inbred lines of maize to the reference inbred B73 and producing 24 RIL families (Buckler *et al.* 2009; McMullen *et al.* 2009). In NAM, 21 starch, 26 protein, and 22 oil QTL were identified, which explained 59, 61, and 70% of the total variation. Of the eight QTL identified in the teosinte NILs, the QTL on chromosomes 1 and 3 appear to be teosinte-specific and were not identified in the NAM (Cook *et al.* 2012) (Figure 4). We identified fewer QTL for kernel starch, protein, and oil in the teosinte NILs. There are multiple nonexclusive reasons that we detected fewer QTL in the NILs compared with NAM: reduced statistical power in NILs as the donor alleles appear at a lower frequency than in RIL populations, and the possible presence of teosinte × teosinte epistatic interactions that are not present in the maize alleles sampled in NAM.

Even though there was a strong correlation between starch and protein at the phenotypic level, only the QTL on chromosome 3 showed complete overlap for starch and protein (as well as oil). The additive effects were strongly negatively correlated (*r* = −0.84, *P* = 0.0045), indicating that this QTL is partially responsible for the high negative correlation between these two traits. This QTL overlap is consistent with the fact that protein and starch are stored primarily in the endosperm and, as a percentage of the kernel, they compensate for each other. In most other cases, however, the QTL appear to be trait-specific. This phenomenon was observed in the NAM population, where several starch and protein QTL colocalized and others did not, despite the similar strong phenotypic correlation (cook *et al.* 2012). It is possible that the chromosome 3 QTL is one of the primary drivers of the 34% increase in starch and 60% loss in protein between teosinte and maize for starch and protein that occurred during domestication (Flint-Garcia *et al.* 2009). However, fine mapping would be required in order to address these questions concerning pleiotropy.

Because our introgressed regions in the teosinte NILs are quite large and the resulting QTL are broad, there are long lists of potential candidate genes underlying each QTL. Rather than dwelling on the possibilities of our favorite candidate genes for which we have little to no supporting evidence at this time, we will focus our discussion on the oil QTL on chromosome 6 that has been identified in many previous QTL studies (Alrefai *et al.* 1995; Zheng *et al.* 2008; Cook *et al.* 2012). The most likely candidate gene is *diacylglycerol acyltransferase 1-2* (*DGAT1-2*), which encodes a rate-limiting enzyme in triacylglycerol biosynthesis that was fine-mapped using NILs and verified by a number of independent methods (Zheng *et al.* 2008). This study identified a 3-bp insertion resulting in an extra phenylalanine residue as the causative lesion conferring the high oil trait. The high-oil insertion allele was present in all 46 teosinte accessions analyzed, and thus the high-oil allele is considered ancestral (Zheng *et al.* 2008). A follow-up study of *DGAT1-2* in landraces and early cycle inbred lines showed the high-oil insertion allele was present in most of the Southwestern US, Northern Flint, and Southern Dent landraces of the United States, at a moderate frequency in Corn Belt Dent, and nearly absent in the early inbred lines (Chai *et al.* 2012). Interestingly, *DGAT1-2* was not identified as a selection candidate by Hufford *et al.* (2012), despite the fact that there were 8–31 SNPs (depending on the definition of gene structure) in *DGAT1-2* in the HapMap2 dataset that could be used for selection tests (Chia *et al.* 2012). One possible reason is the fact that there was no gene model for *DGAT1-2* in B73 RefGen_v1, the version of the genome that was used in the selection study. Alternatively, it is possible that *DGAT1-2* was not selected, but rather the high-oil allele was lost due to drift when the small number of Corn Belt Dent populations was chosen for developing inbred lines as proposed by Chai *et al.* (2012). Regardless of its selection status, it is a strong candidate underlying the chromosome 6 oil QTL.

The teosinte NILs use B73 as the common reference, which allows direct comparisons of the teosinte alleles among themselves, as well as with the NAM inbred founders. Most inbred lines have a lower starch content than B73, thus one might expect that most inbred donor alleles would decrease starch. However, in NAM, of the 132 significant starch alleles, only 82 alleles (62%) decreased starch (Cook *et al.* 2012). In our teosinte NILs, all nine significant alleles decreased starch. In the case of protein, B73 has an average protein content compared with other inbred lines, resulting in an equal mix of significant positive (66 alleles) and negative (69 alleles) effects from the NAM inbred founders (Cook *et al.* 2012). In our study, all 10 of the significant teosinte alleles increased protein content. Interestingly, four of the 27 significant teosinte oil alleles (all from the chromosome 2 QTL) decreased oil, breaking the pattern of expected allelic effects. This is a possible reflection of the smaller difference in oil content between maize and teosinte as compared with the larger differences in protein and starch (Flint-Garcia *et al.* 2009).

The additive effects of the NAM QTL were relatively small with the largest allelic effects being 0.65, −0.38, and 0.21% for the starch, protein, and oil QTL, respectively (Cook *et al.* 2012). We observed that our teosinte alleles were stronger than those of NAM, with the strongest allelic effects of −2.56% for starch, 2.21% for protein, and 0.61% for oil (Table 5). These teosinte alleles may be prime candidates for improving maize kernel composition. The limited number of recombination events in the teosinte NILs compared with the NAM RILs results in larger genomic regions which may contain multiple linked loci that contribute to the larger effects of the teosinte alleles. Unfortunately, there is no set of publicly available NILs carrying a large number of inbred donors (not even the NAM founders) in the B73 background—such NILs would allow comparisons to be made with the exact same population structure. Further, our NIL population was not designed to address this question without initiating fine-mapping experiments for the various alleles. However, we have developed a different population with a higher proportion of the same teosinte donors and more extensive recombination to further address this question (S.A. Flint-Garcia, unpublished data).

The maize teosinte NILs were developed to reintroduce a modest amount of genetic variation (∼3% teosinte donor on average) from teosinte and evaluate the value of teosinte alleles for various agronomic and kernel composition traits (Liu *et al.* 2016). In this study, we determined the genetic basis of kernel composition alleles from teosinte, and compared the QTL and their effects to those observed in in the maize NAM population. We identified teosinte alleles with a broader range and larger allelic effect in comparison to that observed in diverse maize. Our study strongly suggests that teosinte bears novel alleles that can be utilized for the improvement of kernel starch, protein, and oil content in modern maize germplasm, as well as provide unique source of variation for further QTL and molecular studies.

## Supplementary Material

Supplemental material is available online at www.g3journal.org/lookup/suppl/doi:10.1534/g3.117.039529/-/DC1.
